# Resistance of (*Aegilops tauschii* × *Secale cereale*) × *Triticosecale* Hybrids to Leaf Rust (*Puccinia triticina*) Determined on the Macroscopic and Microscopic Level

**DOI:** 10.3389/fpls.2018.01418

**Published:** 2018-09-26

**Authors:** Maciej Majka, Albrecht Serfling, Paweł Czembor, Aurelia Ślusarkiewicz-Jarzina, Michał Tomasz Kwiatek, Frank Ordon, Halina Wiśniewska

**Affiliations:** ^1^Department of Genomics, Institute of Plant Genetics, Polish Academy of Sciences, Poznań, Poland; ^2^Institute for Resistance Research and Stress Tolerance, Julius Kühn Institute, Federal Research Centre for Cultivated Plants, Quedlinburg, Germany; ^3^Department of Genetics and Plant Breeding, Plant Breeding and Acclimatization Institute – National Research Institute, Błonie, Poland; ^4^Department of Biotechnology, Institute of Plant Genetics, Polish Academy of Sciences, Poznań, Poland

**Keywords:** adult plant resistance, *Aegilops tauschii*, doubled haploids, leaf rust, marker-assisted selection, *Puccinia triticina*, seedling resistance, triticale

## Abstract

Leaf rust caused by *Puccinia triticina* Eriks belongs to the most important fungal pathogens of wheat (*Triticum aestivum* L.) and triticale (× *Triticosecale*). Effective resistance to leaf rust is both, cost-effective and environmentally safe. Many wild *Aegilops* species carry unknown resistances against fungal diseases and are characterized by a high genetic variability. The main goal of this work was to examine the resistance of (*Aegilops tauschii* × *Secale cereale*) × *Triticosecale* hybrids to leaf rust in inoculation tests with different races of *P*. *triticina*. Hybrid plants were selected for the presence of 2D chromosome/s in the triticale background using fluorescence and genomic *in situ* hybridization. The presence of leaf rust resistance genes was confirmed with closely linked molecular markers, i.e., *Xgdm35* and *Xgwm296*. 14 genotypes of BC_2_F_4_ – BC_2_F_6_ hybrid plants with the monosomic addition of chromosome 2D (M2DA) were analyzed together with nine control lines. Resistance was determined at the macroscopic and microscopic level at the seedling and adult plant stage (flag leaf). In general, results revealed limited resistance of hybrid plants at the seedling stage, followed by an increase of the resistance level at later stages of plant development. This indicates that respective hybrid plants may exhibit APR resistance conferred by *Lr22a* introgressed from *Ae*. *tauschii*. On the basis of the macroscopic and microscopic analysis, this kind of resistance turned out to be additive and race-specific. We selected four monosomic 2D addition triticale genotypes highly resistant to *P*. *triticina* infection at the two main stages of plant development. From the selected genotypes, we obtained 26 doubled haploid lines among which two lines with doubled additional chromosomes 2D of *Ae*. *tauschii* can be used for further breeding to increase leaf rust resistance of cultivated triticale.

## Introduction

Leaf rust caused by *Puccinia triticina* Eriks, is one of the most destructive and important foliar diseases of cereals, especially because of the wide distribution and potential to develop rapidly under optimal environmental conditions (Kolmer, 1996; Dean et al., 2012; Chen et al., 2013). Yield losses caused by *P*. *triticina* range between 10 up to 70% (Roelfs et al., 1992; Huerta-Espino et al., 2011). Infections result in a reduction of kernels per ear, lower kernel weight and degradation in grain quality. In order to avoid yield losses and reduced quality, host resistance is both cost-effective and environmentally safe (Assefa and Fehrmann, 2000; Kalia et al., 2017).

Genetic resistance to leaf rust can be determined as seedling resistance or adult plant resistance (APR). Seedling resistance, also defined as major gene, race-specific or qualitative resistance confers a high level of resistance mostly caused by a hypersensitive response. The APR is described as a susceptible reaction at the seedling stage, followed by an increased level of resistance in further stages of plant development. APR can be either race-specific (e.g., *Lr12*) or race-non-specific (e.g., *Lr34*) and is usually measured on the flag leaf (Dyck and Kerber, 1985; Park and McIntosh, 1994; McIntosh et al., 1995).

Wild *Aegilops* species constitute a rich source of genetic variability of agronomically important traits (Frankel and Bennett, 1970), especially resistance to diseases (leaf/brown rust, yellow rust, stripe rust and powdery mildew) (Valkoun et al., 1985; Gill et al., 1986; Cox et al., 1992; Assefa and Fehrmann, 2004). Due to the close genetic relationship with cereals, goat grasses have been used successfully in breeding programs conducted in order to improve the triticale (× *Triticosecale* Wittm.) and wheat (*Triticum aestivum* L.) using conventional crossing and recombination methods (Schneider et al., 2008; Apolinarska et al., 2010).

A number of leaf rust resistance genes were transferred from *Ae*. *tauschii* to cultivated wheat. *Ae*. *tauschii* is the source of seedling resistance genes *Lr21* (1D), *Lr32* (3D), *Lr39* (2D), *Lr42* (1D), and APR gene *Lr22a* (2D) (Rowland and Kerber, 1974; Kerber, 1987; Cox et al., 1994; Raupp et al., 2001). *Lr22a* confers resistance at the adult plant stage comparable to the resistance conferred by seedling resistance genes, contrary to the slow-rusting type APR determined by genes, e.g., *Lr34* (Lagudah et al., 2006). Furthermore, the absence of virulence to *Lr22a* is partially explained by its lack of exposure to pathogen, thus this gene can be used for pyramiding of leaf rust resistance (McIntosh et al., 1995; Hiebert et al., 2007). In the United States, there are no reports of cultivars carrying this gene (Kolmer, personal communication). Using TACCA (targeted chromosome-based cloning via long-range assembly) Thind et al. (2017) cloned the broad-spectrum of *Lr22a* and found that this gene encodes an intracellular immune receptor homologous to the RPM1 protein of *Arabidopsis thaliana*. Another resistance gene on the short arm of chromosome 2D is *Lr39* which has been transferred already to modern cultivars, however, virulent races were observed already in some regions of the world (Kolmer and Hughes, 2017).

The combination of both seedling and APR genes on chromosome 2D together with genes determining agronomically important traits (Jia et al., 2013), indicates that this chromosome is best suited for improving Triticeae species by wide crosses. *Ae*. *tauschii* × *Secale cereale* amphiploids, as a bridge between wild and cultivated species, constitute a successful way to transfer the D-genome chromatin into triticale. Utilization of this amphiploids allowed Kwiatek et al. (2015) to transfer the 3D chromosome carrying *Lr32* from *Ae*. *tauschii* into triticale, whereas Majka et al. (2016) obtained monosomic addition lines of triticale carrying chromosome 2D of *Ae*. *tauschii* (M2DA), which let to changes in plant height, spike morphology and an increased resistance to *Blumeria graminis*.

The acceleration of breeding progress is one of the crucial matters for crop improvement. Doubled haploids (DHs) directly leading to homozygous plants, considerably shortened the breeding process in many inbreeding plant species (Murovec and Bohanec, 2012). In triticale DHs are usually obtained by androgenesis induced in *in vitro* anther cultures and are routinely used in triticale breeding (Ślusarkiewicz-Jarzina and Ponitka, 1997, 2003; Ślusarkiewicz-Jarzina et al., 2017).

The main goal of this work was to examine the resistance of (*Ae*. *tauschii* × *S*. *cereale*) × *Triticosecale* hybrids to leaf rust. For that purpose, inoculation with variable isolates of *P. triticina* carrying different virulence patterns was performed, to get information on genes transferred from *Ae. tauschii*. In order to obtain stable triticale lines with introgression of D-genome chromatin and with resistance to leaf rust, DHs of selected resistant hybrids were produced and analyzed. To achieve this macroscopic and microscopic analyses of infection were conducted complemented by molecular marker analyses and cytogenetic examination of hybrid plants.

## Materials and Methods

### Plant Material and Growing Conditions

Seeds of *Aegilops tauschii* Coss. (D51; 2*n* = 2*x* = 14; DD), *Secale cereale* L. (Strzekecinskie; 2*n* = 2*x* = 14, RR) and × *Triticosecale* Wittm. (Bogo; 2*n* = 6*x* = 42; AABBRR) originating from the collection of the Institute of Plant Genetics, PAS, were used in this study. The *Ae*. *tauschii* × *S*. *cereale* amphiploids (2*n* = 4*x* = 28; DDRR) were obtained using embryo rescue by Sulinowski and Wojciechowska in the IPG PAS (data unpublished). The F_1_ (*Ae*. *tauschii* × *S*. *cereale*) × triticale hybrids were obtained by crossing of triticale cv. Bogo with *Ae*. *tauschii* × *S*. *cereale* amphiploids as a pollinator. Backcrosses with the triticale as a male parent were used to achieve the following generations (BC_1_F_1_ and BC_2_F_1_) and further selfed to produce the BC_2_F_4_ to BC_2_F_7_ hybrid plants. Seeds of *T*. *aestivum* L. (Thatcher and Borenos, 2*n* = 6*x* = 42; AABBDD), near-isogenic lines (NILs) of wheat cv. Thatcher, i.e., Tc + *Lr22a* and Tc + *Lr39* originated from the collection of the Julius Kuehn Institute (JKI) and the Plant Breeding and Acclimatization Institute – National Research Institute (PBAI-NRI). Cultivar Thatcher and Borenos were used as standards susceptible to leaf rust. Cultivation was conducted at 80% ± 10% humidity, at 20°C ± 2°C and a light intensity higher than 300 ± 15 mmol under daylight conditions (16 h) on the level of the soil surface.

### Chromosome Preparation and Labeling of Probes

Germination, metaphase accumulation, and fixation procedures were carried out according to Kwiatek et al. (2016a). The chromosome preparations were made from root tips using the squash method according to Hasterok et al. (2006) with minor modifications. Three repetitive sequences were used for FISH: pTa-535, pTa-86 and pTa-k374 which were amplified and labeled according to Kwiatek et al. (2016b). Probe pTa-535 was labeled using tetramethyl-5-dUTP-rhodamine (Roche), pTa-86 was labeled with digoxigenin-11-dUTP (Roche) and pTa-k374 with Atto647N (Jena BioScience). Total genomic DNA was extracted from fresh leaves of *Ae. tauschii* (DD) and triticale ‘Bogo’ (AABBRR) using GeneMATRIX Plant and Fungi DNA Purification Kit (EURx). In GISH experiments, genomic DNA from *Ae. tauschii* was labeled with Atto647N (Jena BioScience). Blocking DNA from triticale was sheared to fragments of 5–10 kb by boiling for 30–45 min and used at a ratio of 1:50 (probe:block).

### Fluorescent and Genomic *in situ* Hybridization (FISH and GISH)

FISH and GISH procedures were performed according to Kwiatek et al. (2016a) with minor modifications. Three probes (pTa-535, pTa-86, pTa-k347) were subjected to FISH on the same chromosome preparations. The hybridization mixture (20 μl per slide) contained 90 ng of each probe in the presence of salmon sperm DNA, 50% formamide, 2 × SSC, and 10% dextran sulfate, and was denatured at 70°C for 10 min and stored on ice for 5 min. Chromosomal DNA was denatured in the presence of the hybridization mixture at 70°C for 3 min on a heating table (Medax) and allowed to hybridize overnight at 37°C. Digoxigenin detection was made using anti-digoxigenin-fluorescein antibody (Roche). After documentation of the FISH sites, the slides were washed according to the procedure of Heslop-Harrison (2000) with minor modifications (2 × 15 min in 4 × SSC + 0.2% Tween at 37°C and 1 × 5 min in 2 × SSC, at room temperature). GISH, with total genomic DNA of *Ae*. *tauschii* as a probe and DNA of triticale as a block, was made with the same conditions after reprobing. Ten metaphases per slide were analyzed and documented with the Olympus BX61 automatic epifluorescence microscope supplied with an Olympus XM10 CCD camera. Image processing was carried out using the Olympus Cell-F imaging software (version 3.1; Olympus Soft Imaging Solutions) and PaintShop ProX5 software (version 15.0.0.183; Corel Corporation). The identification of particular chromosomes was made in reference to previous reports of Cuadrado and Jouve (1994) and Komuro et al. (2013).

### PCR Amplification of Molecular Markers

Genomic DNA was extracted from fresh leaves of single genotypes of (*Ae*. *tauschii* × *S*. *cereale*) × *Triticosecale* hybrid plants as well as positive and negative controls: *Ae*. *tauschii*, triticale cv. Bogo, *Ae*. *tauschii* × *S*. *cereale* amphiploid, wheat cv. Thatcher and NILs: Tc + *Lr22a* and Tc + *Lr39*, using GeneMATRIX Plant and Fungi DNA Purification Kit (EURx). Two molecular markers localized on chromosome 2D were used: *Xgdm35* and *Xgwm296* (Roder et al., 1998; Pestsova et al., 2000). PCR reactions were performed according to Majka et al. (2017) with appropriate annealing temperature (52–60°C). Amplification products were separated in agarose gels (Sigma-Aldrich), stained, visualized and photographed according to Majka et al. (2017).

### Isolates of *P*. *triticina*, Inoculation of Plants and Infection Ratings

For the analyses of hybrids resistance to leaf rust infection, three isolates of *P*. *triticina* from the JKI collection were used. Isolate 1 one was the aggressive isolate (wxr77), has a high germination rate and generates the highest number of uredospores per pustule on susceptible genotypes in comparison to all other isolates used for experiments. The isolate is avirulent against *Lr22a* at the adult stage and develops uredospore pustules delayed at the seedling stage. Furthermore isolate 1 is avirulent to *Lr39*. Isolate 2 was virulent for *Lr22a* at all stages and isolate 3 was virulent to *Lr39* (resulting in infection rate from 2 to 3) at all stages of plant development. In order to find virulence/avirulence pattern useful for plant genotypes tested at PBAI-NRI we include for initial screening 10 isolates originating from wheat (Pt13-4-1, Pt14-82-1, Pt14-84-1, Pt14-85-1, Pt15-82, Pt15-89, Pt15-94-1, Pt15-100, Pt15-101, Pt15-103) and one from triticale (Pt11WT12). These isolates were chosen from the PBAI-NRI collection based on host origin and diverse response of Thatcher NILs containing *Lr22a* and *Lr39* genes (data not shown). Initial screening of isolates was performed at seedling and adult plant stage on five genotypes: *Ae*. *tauschii*, triticale cv. Bogo, wheat cv. Thatcher and two NILs of that cultivar with leaf rust resistance genes Tc + *Lr22a* and Tc + *Lr39*.

For the analysis of the level of resistance (JKI), five plants of each genotype were planted in pots with a diameter of 5.5 cm in four replications in soil for germination (Archut-Fruhstorfer Erde, Hawita). Seedlings (first leaf fully expanded) were inoculated 12 days after germination with 10mg of leaf rust uredospores mixed with 10 mg of dry powdered clay per pot using a settling tower (Isolates 1–3) (Hoogkamp et al., 1998). In the experiments with isolates from PBAI-NRI collection, 10-day-old seedlings (3–5 per genotype) grown in 8 × 13-cell horticultural plastic tray (30 cm × 60 cm) filled with pindstrup mix special substrate (Pindstrup) were inoculated with 80 mg of uredospores, using the spraying method (Roelfs et al., 1992). In all tests, inoculated plants were kept in a humid glass chamber at 25°C for incubation for the first 24 h and then, shifted to a muslin cloth chamber under 24°C/20°C (16-h day/8-h night) regime.

After tests at seedling stage all genotypes planned for inoculation at adult plant stage (flag leaf) were grown at 4°C/8 weeks for vernalization and preservation of plants at the same plant growth stage as possible. After this period each seedling was planted to individual larger pot (20 cm × 20 cm × 30 cm depth) filled with pindstrup mix special substrate and kept at glasshouse (at least 16 h light and temperature below 28°C) until testing flag leaf. Nevertheless, because of different maturity, the genotypes were tested in two series, so plants had flag leaf developed at the same stage. Plants with fully developed flag leaves were moved back to plant growth chamber using temperature and humidity for inoculation and incubation period as described earlier. However, flag leaves of adult plants were inoculated using uredospores in rate 200 mg/50 ml water/24 pots.

Disease reaction (both, seedlings and flag leaves) was recorded for all accessions and different isolates at 8–10 days after the inoculation using 0–4 scale described by McIntosh et al. (1995). This rating system allows the classification as “immune” (rated as “0”), “very resistant” (rated as “;” or “0.5”), “resistant” (rated as “1”), “moderately resistant” (rated as “2”) “moderately resistant to moderately susceptible” (rated as “3”) and “susceptible” (rated as “4”) to leaf rust. In parallel genotypes were vernalized for 8 weeks (4°C) before infection at the flag leaf stage in order to analyze resistance reaction of adult plants.

### Staining Procedures and Microscopy

Leaves of inoculated plants were collected at 48 hai (hours after inoculation), 72 hai, 96 hai, and 168 hai by cutting leaves to segments of 1.5 cm length. Segments were incubated in reaction tubes (2 ml) in 1.5 ml of the 3,3′ Diaminobenzidine (DAB) solution for 16 h at room temperature. Fungal cell walls were stained with Calcofluor White M2R (Rohringer et al., 1977) according to Serfling et al. (2016). Samples were washed with sterile water, transferred to a microscope slide and embedded in a glycerol/water solution (1:1 v/v). Microscopy of leaf cells and fungal structures was performed using a Zeiss Axioskop 50 microscope supplied with the AxiocamMRc camera connected with the software package Axiovision 4 (Carl Zeiss). Fungal structures were observed using the filter set 02 (excitation filter G 365, beam splitter FT 395, and barrier filter LP 420), autofluorescence within plant tissue was observed using the filter set 05 (excitation filter BP 400-440, beam splitter FT 460, barrier filter LP 470). These conditions enabled the visualization of pathogen development, i.e., germinating spores, appressoria and haustorial mother cells (HMCs). This procedure also allowed the detection of host cell micronecrosis autofluorescence adjacent to HMC. For experiments in which fungal structures (hmc) were counted, four leaf segments from the middle of the youngest leaf of four seedlings from four different plants of the same genotype were microscopically analyzed. Within each replication 10 infection sites (germinated uredospore and appressorium generated) were counted, thus counts of 40 infection sites (4 × 10) were used for the analysis.

### Doubled Haploid Production

Tillers from the monosomic addition lines of triticale with chromosome 2D were cut at the uninuclear stage of the microspore development and cold treated at 4°C for 6 days in the mineral salt medium N_6_ (Chu et al., 1975) in the dark before plating the anthers. Anthers were cultured on the C17 medium (Wang and Chen, 1983) with 90 g/l maltose (instead of sucrose), 2.0 mg/l 2,4-D and 0.5 mg/l KIN in the dark at 28°C for 4 weeks. The androgenic embryos developed from the microspores were transferred to the regeneration medium 190-2 with 1.0 mg/l KIN, 0.5 mg/l NAA and 30 g/l saccharose (Zhuang and Xu, 1983). Organogenesis was induced at 22°C in the continuous light for 12 h per day. The ploidy levels of green plants were determined by flow cytometry. The identified haploid plants were subjected to colchicine treatment (0.1% aqueous solution with 4% DMSO and 25 mg/l GA_3_) for 6 h in light at 25°C. After the treatments, plants were washed, potted, vernalized for 6 weeks at 4°C and moved to the greenhouses where they were grown to maturity.

### Statistical Analysis

The macroscopic and microscopic observations data were subjected to statistical analysis. Two-way analysis of variance (ANOVA) with a homogenous groups of genotypes (hybrids and controls) and stage of development (seedling and adult plants) as classification factors and Fisher’s least significant difference test (LSD) were made using STATISTICA 10 software (StatSoft, Tulsa, OK, United States). Fisher’s LSD of samples at 5% was used.

## Results

### Identification of Monosomic 2D Addition Hexaploid Triticale Plants

**FIGURE 1:**
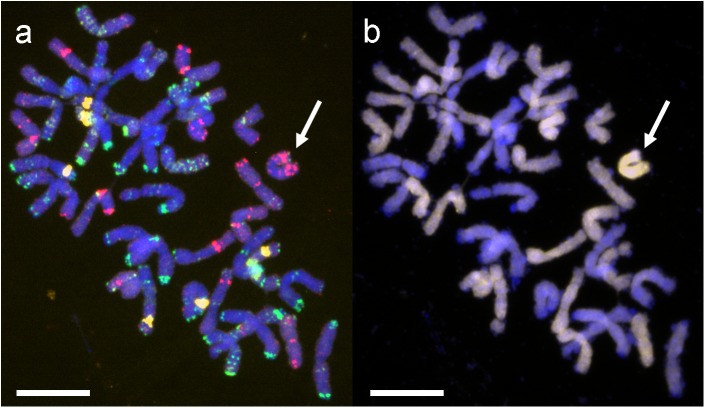
Mitotic chromosomes of hybrid genotype 1 (BC_2_F_4_) (*Ae*. *tauschii* × *S*. *cereale*) × *Triticosecale* cv. Bogo analyzed using **(a)** FISH with pTa-535 (red), pTa-86 (green) and pTa-k374 (28S rDNA) (yellow) probes, **(b)** GISH with total genomic DNA probe of *Ae*. *tauschii* (D, yellow) and triticale (ABR, blocking DNA). Arrows indicate the introgressed chromosome 2D. Scale bars 10 μm.

The chromosome composition of (*Ae. tauschii* × *S. cereale*) × triticale cv. Bogo hybrids was verified using FISH and GISH. In FISH analyses three BAC clones were mapped: pTa-k374 (28S rDNA), pTa-535 (pAs1 related), pTa-86 (pSc119) (**Figure 1a**), and in GISH D-genome probe (DNA of *Ae*. *tauschii*) and blocking DNA from triticale were applied (**Figure 1b**). Because of the diversity of genes and according to previous results (Majka et al., 2016), hybrid plants were selected for the presence of 2D chromosome/s in triticale background. Fourteen genotypes of BC_2_F_4_ – BC_2_F_6_ hybrid plants with the monosomic addition of chromosome 2D (M2DA) were used. Additionally, two genotypes of BC_2_F_7_ without *Lr* genes, but carrying one 2D chromosome and pair of 2D chromosomes were chosen as susceptible controls. As a complement, seven control lines were applied: *Ae*. *tauschii, Ae*. *tauschii* × *S*. *cereale* amphiploid form, triticale cv. Bogo, wheat cv. Borenos and Thatcher as well as two NILs: Tc + *Lr22a* and Tc + *Lr39* (**Supplementary Tables S1, S2**). Plant material was divided into three groups. The first group was designated for the macroscopic and microscopic analysis of the leaf rust resistance performed in JKI, the second group for the macroscopic analysis of the leaf rust resistance performed in PBAI-NRI and a third group of hybrid plants for the DH production (IPG PAS).

### PCR Analysis of Markers Related to Leaf Rust Resistance Genes

In this study, the presence of two molecular markers associated with the leaf rust resistance genes, i.e., *Xgwm296* and *Xgdm35* (*Lr22a* and *Lr39*, respectively) localized on chromosome 2D was verified (Roder et al., 1998; Pestsova et al., 2000). Results obtained for *Xgdm35* marker revealed that the allele linked to resistance (PCR product of 180 bp) was present in all analyzed genotypes of hybrid plants and control plants: *Ae*. *tauschii*, amphiploid form *Ae*. *tauschii* × *S*. *cereale* and Tc + *Lr39*. We observed products of different size (indicative for susceptibility) in two genotypes of hybrid plants with one 2D chromosome and a pair of 2D chromosomes which were selected as control plants without resistance genes as well as for triticale Bogo and Tc + *Lr22a*. The size of the bands differed between *Ae*. *tauschii* and NILs of *T*. *aestivum* cv. Thatcher with *Lr39*. The presence of *Lr22* was determined with *Xgwm296* marker. The banding pattern specific for resistance allele *Lr22a* (120 + 150 bp) was present in all analyzed genotypes of hybrid plants and control plants: *Ae*. *tauschii*, amphiploid form *Ae*. *tauschii* × *S*. *cereale* and Tc + *Lr22a*.

### Selection of the Isolates

**FIGURE 2:**
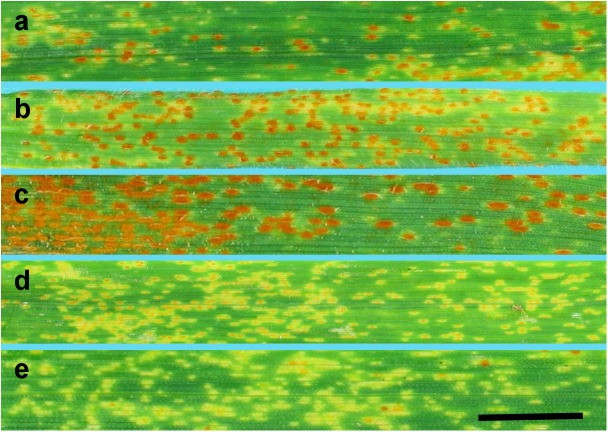
Macroscopic observation of the infection rates (8 days) after inoculation of control plants with Pt11WT12 (isolate 4) at the adult plant stage (flag leaf). Wheat cv. Thatcher (rated as “3”) **(a)**, *Ae*. *tauschii* (rated as “3”) **(b)**, triticale cv. Bogo (rated as “4”) **(c)**, Tc + *Lr22a* (rated as “2”) **(d)**, Tc + *Lr39* (rated as “1”) **(e)**. Scale bar 1 cm.

To select appropriate isolate with virulence/avirulence pattern useful for plant genotypes tested at PBAI-NRI, 11 isolates of *P*. *triticina* from the PBAI-NRI collection were tested at seedling (first leaf) and adult plant stage (flag leaf) (**Table 1** and **Figure 2**). From among isolates tested, Pt11WT12 was chosen for further studies, since this isolate was the only virulent to triticale cv. Bogo at both plant growth stages and could differentiate Thatcher lines with *Lr22a* and *Lr39*.

**Table 1:** Reaction of plant genotypes (scale 0–4, description in “Materials and Methods” section) at seedling and adult plant stage to inoculation with 11 isolates of *P*. *triticina*.

Isolate	Seedlings (first leaf)					Adult plants (flag leaf)				
		
	Thatcher (Tc)	*Ae*. *tauschii*	Bogo	Tc + *Lr22*	Tc + *Lr39*	Thatcher (Tc)	*Ae*. *tauschii*	Bogo	Tc + *Lr22*	Tc + *Lr39*
Pt13-4	4	4	0	4	2	4	4	0	1	2
Pt14-82	4	4	0	4	2	4	4	0	0	0
Pt14-84	4	4	0	4	1	4	4	0	2	1
Pt14-85	4	4	0	4	2	4	4	0	3	3
Pt15-100	4	4	0	4	3	4	4	0	0	0
Pt15-101	4	4	0	4	2	4	4	0	0	0
Pt15-94	4	4	4	4	3	4	4	0	1	2
Pt15-89	4	4	0	3	2	4	4	0	0	0
Pt15-82	4	4	0	4	2	4	4	0	1	0
Pt11WT12	4	4	4	3	2	3	3	4	2	1
Pt15-103	4	4	0	4	3	N/A	N/A	N/A	N/A	N/A


### Macroscopic Analysis of Leaf Rust Infection

**FIGURE 3:**
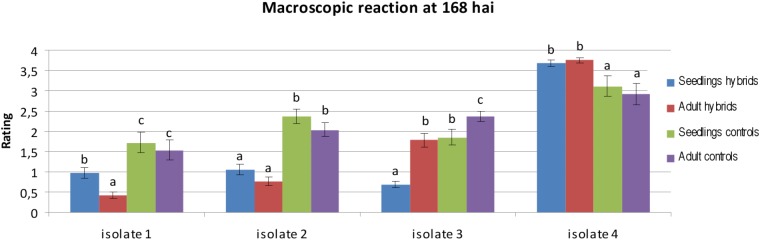
Statistical analysis of macroscopic reaction of hybrids carrying leaf rust resistance genes (hybrids) and control plants (controls) inoculated with single spore isolates at 168 hai at the seedling and adult plant stage. The bars represent a mean value (over replications) and error bars represent standard errors. The letters indicate homogenous groups of means that do not differ significantly at a significance level of 0.05 (Fisher’s LSD-test).

**FIGURE 4:**
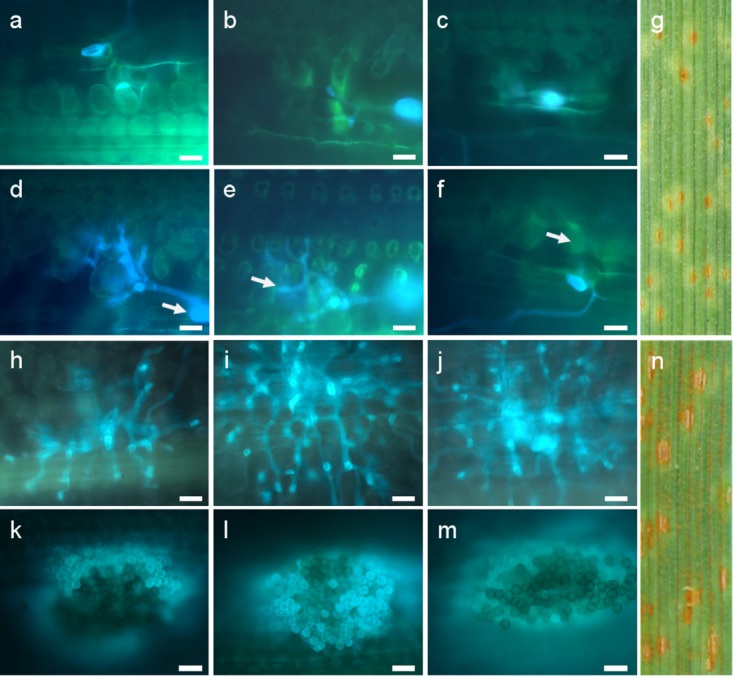
Development of fungal structures in the resistant hybrid genotype 1 **(a–f)** and the susceptible hybrid genotype 12 **(h–m)** after inoculation with isolate 1 **(a,d,h,k)**, isolate 2 **(b,e,i,l)**, and isolate 3 **(c,f,j,m)** at the seedling stage. Macroscopic symptoms on leaf segments 8 days after inoculation with isolate 4 **(g,n)** and microscopic observations 72 hai **(a,b,c,h,i,j)** and 168 hai **(d,e,f,k,l,m)** are compared. Arrows indicate substomatal vesicle **(d)**, haustorial mother cell **(e)**, and autofluorescence around infection sites **(f)**. Scale bars 20 μm.

The macroscopic evaluation of infection rates was performed 8 days after inoculation for three isolates from the JKI collection (Isolates 1–3), and one isolate from the collection of PBAI-NRI (Isolate 4) for seedlings and adult plants (**Supplementary Tables S1, S2**). On the basis of two-way ANOVA we observed significant differences at *P*-value lower than 0.05, between classification factors, i.e., a homogenous groups of genotypes (hybrids and controls) and stage of development (seedling and adult plants). In case of homogenous groups of genotypes we observed significant differences for all four isolates. We identified significant differences between stage of development for isolates 1, 2, and 3. ANOVA did not show significant differences in the interaction between homogenous groups of genotypes and stage of development. The infection rate of isolates 1 and 2 was higher at the seedling stage in comparison with the adult plant stage (mean 0.98–0.43 for isolate 1 and 1.05–0.76 for isolate 2 in hybrid plants). The same was observed for isolate 3 at the adult plant stage (mean 0.69–1.79 in hybrid plants). Isolate 4 was the most aggressive (mean 3.02 for hybrids), whereas isolate 1 was the least aggressive for all genotypes (mean 0.70 for hybrids) (**Figure 3**). The selection of the most resistant genotypes on the basis of macroscopic rates, was difficult, because of the similarities of infection rates obtained. Only in case of isolate 4, four genotypes with a lower rate (3 instead of 4) at both analyzed stages of development were selected: Hybrid genotype 1 (**Figure 4g**), Hybrid genotype 2, Hybrid genotype 11 and Hybrid genotype 14. The reduced infection rate was identified also for *Ae*. *tauschii*, amphiploids (*Ae tauschii × S*. *cereale*) and the two NILs (**Supplementary Tables S1, S2**). *Ae*. *tauschii* plants were characterized by high infection rates at both stages and for all used isolates (**Supplementary Tables S1, S2**). For amphiploid forms, we identified a similar level of infection in comparison to the mean infection rate obtained for hybrid plants, however, in case of isolate 4, amphiploids were less infected (**Figure 3** and **Supplementary Tables S1, S2**). The rating of triticale cv. Bogo revealed no significant differences to the hybrids. We observed no dependence between generations of hybrid plants and resistance. However, among four outstanding genotypes with the higher resistance, two of them were from the earliest generation BC_2_F_4_. Infection rates established for adult control plants were lower in comparison to the infection rates evaluated at the seedling stage for this group (**Figure 3** and **Supplementary Tables S1, S2**).

### Microscopic Analysis of Fungal Structures Development

The infection level was determined on the basis of the number of HMCs at 72 hai and colonies with uredospore pustules per mm^-2^, 7 days after inoculation. Resistance level was estimated for seedlings and adult plants (**Supplementary Tables S1, S2**, respectively).

**FIGURE 5:**
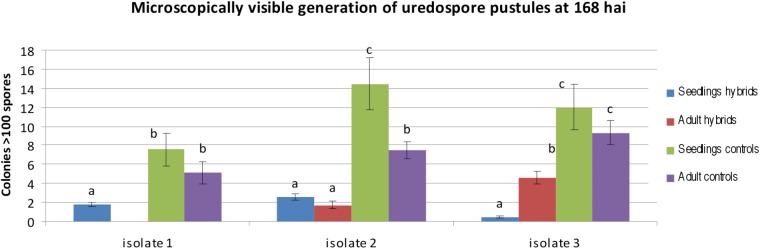
Statistical analysis of microscopically visible generation of uredospore pustules of hybrids carrying leaf rust resistance genes (hybrids) and control plants (controls) inoculated with single spore isolates at 168 hai at the seedling and adult plant stage. The bars represent a mean value (over replications) and error bars represent standard errors. The letters indicate homogenous groups of means that do not differ significantly at a significance level of 0.05 (Fisher’s LSD-test).

On the basis of two-way ANOVA we observed significant differences at P-value lower than 0.05, between classification factors, i.e., a homogenous groups of genotypes (hybrids and controls) and stage of development (seedling and adult plants). In case of homogenous groups of genotypes we observed significant differences for all three isolates. We identified significant differences between stage of development for isolates 1 and 2. ANOVA show significant differences in the interaction between homogenous groups of genotypes and stage of development for isolates 2 and 3. Obtained results revealed that hybrids and control plants at the adult plant stage were less infected than seedlings in case of isolates 1 and 2 (mean 0.00 to 1.81 for isolate 1 and 1.71 to 2.60 for isolate 2 in hybrid plants), whereas after inoculation with isolate 1 we identified no infection on the hybrid plants (**Supplementary Table S1** and **Figure 5**). Hybrid genotype 1 (**Figures 4a–f**), Hybrid genotype 2, Hybrid genotype 11, and Hybrid genotype 14 were very resistant and Hybrid genotype 12 (**Figures 4h–n**) very susceptible to all three isolates at both stages of development (**Supplementary Tables S1, S2**). Two of the most resistant genotypes were from the earliest generation – BC_2_F_4_. Triticale cv. Bogo and the amphiploid *Ae*. *tauschii* × *S*. *cereale* were very resistant in contrast to *Ae*. *tauschii* and both susceptible standard wheat cultivars, i.e., Thatcher and Borenos. The number of HMC and uredospores in two genotypes with introgression of 2D chromatin but without resistance genes were similar to those obtained for triticale. Tc + *Lr22a* plants were more infected at the seedling stage than at the adult plant stage, especially in case of inoculation with isolate 2 (**Supplementary Tables S1, S2**). What is more, adult control plants were less infected than seedlings for all isolates (**Figure 5**).

### Production of DH Lines

A total of 5,634 anthers collected from three M2DA triticale plants (Hybrid genotype 1, 2, and 11) were used to produce 2,271 androgenic structures. The lowest number of androgenic structures (427) was obtained from the anthers of plant M2DA_1 (Hybrid genotype 1), followed by 796 androgenic structures obtained from plant M2DA_2 (Hybrid genotype 2), whereas the highest number of those structures (1,048) was obtained from anthers of plant M2DA_3 (Hybrid genotype 11). Overall, 61 haploid plants were obtained, of which 36 were generated from plant M2DA_3. After the colchicine treatment, 26 DH plants were generated, which were self-pollinated and harvested (**Table 2**). Flow cytometry experiments confirmed the ploidy level of androgenic plants, derived DH plants and control triticale cv. “Bogo.” The efficiency of androgenesis is presented in **Table 2**, whereas numbers of spikes and seeds of DH lines are presented in **Table 3**. We obtained 26 lines of DH among which all plants of two lines were characterized by the presence of two 2D chromosomes in the triticale background. One line was derived from the Hybrid genotype 1 and second line from the Hybrid genotype 2.

**Table 2:** The effectiveness of androgenesis induction and doubled haploid production from three monosomic 2D addition plants of triticale.

Plant	Number of anthers	Androgenic structures		Green plants		Plants for colchicine treatment	Haploids		Doubled haploids		Aneuploids	
						
		Number	Per 100 anthers	Number	Per 100 anthers		Number	%	Number	%	Number	%
M2DA_1	1613	427	26.5	8	0.5	3	2	66.7	1	33.3	0	0
M2DA_2	1704	796	46.7	17	1.0	11	7	63.6	4	36.4	0	0
M2DA_3	2317	1048	45.2	36	1.6	34	13	38.2	21	61.8	0	0


**Table 3:** Characterization of androgenesis induction and doubled haploid production from three monosomic 2D addition plant of triticale.

Plant	DH lines					
	
	Total number	Chromosome constitution	Number of lines	Number of spikes	Number of seeds	Number of analyzed plants
M2DA_1	1	42T + D2D	1	5	105	50
M2DA_2	4	42T + D2D	1	3	61	50
		42T	3	14	572	50
M2DA_3	21	42T	21	130	3239	50


## Discussion

*Ae*. *tauschii* is an excellent source of genes that could be used in breeding of Triticeae species, especially determining resistance to fungal diseases like leaf rust (Valkoun et al., 1985; Gill et al., 1986; Dhaliwal et al., 1991). The main goal of this work was to examine the resistance of (*Ae*. *tauschii* × *S*. *cereale*) × *Triticosecale* hybrids to leaf rust in inoculation tests with isolates of *P. triticina* at two stages of plant development. Macroscopic and microscopic analysis of infection were combined with molecular markers associated with leaf rust resistance genes and cytogenetic analysis of hybrid plants. Selected resistant genotypes were stabilized by the production of double haploids.

Allocation of chromosome 2D of *Ae*. *tauschii* (D51) in triticale background leads to the expression of genes determining resistance to powdery mildew, semi-dwarfism and spike morphology (Majka et al., 2016), whereas Kwiatek et al. (2015) reported a positive transfer of 3D chromosomes with *Lr32* from *Ae*. *tauschii* into triticale cv. Bogo. The use of this wild species as a source of leaf rust resistance genes was confirmed by Majka et al. (2017). Besides the fact that triticale cv. Bogo was determined as a cultivar very resistant to leaf rust infection (Grzesik and Strzembicka, 2003), this resistance may break down in the next few years. In this respect, genotypes that carry complex disease resistance are most valuable. Thus, pyramidization of triticale resistance with seedling and APR resistance from *Ae*. *tauschii* would play a key role for the obtaining durable resistance in this species.

The exact resistance of triticale hybrids with the introgression of 2D chromosomes was determined in the inoculation tests, which were performed on seedlings and adult plants. The selection of isolates according to macroscopic observations of infection rates, showed that triticale cv. Bogo is very resistant (rate 0) to leaf rust (10/11 isolates) at both stages of development (**Table 1**) in accordance with previous reports (Grzesik and Strzembicka, 2003). Grzesik and Strzembicka (2003) revealed that in greenhouse and field trials, this cultivar was highly resistant to both isolates (65a/95 and 81c/95) used for inoculation. Only one isolate (isolate 4; Pt11WT12) undoubtedly was virulent to triticale cv. Bogo (rate 4) (**Table 1**). Inoculations of *Ae*. *tauschii* with *P. triticina* isolates demonstrated a high level of infection. It was very surprising because this species was believed as a source of leaf rust resistance, but in exceptional cases, like dry and cold winters, this species may not have seedling resistance (Kalia et al., 2017). In this work, the susceptibility of *Ae*. *tauschii* to all analyzed isolates of *P*. *triticina* may result from differences between the origin of this species and the origin of the isolates. It was reported that the *Ae*. *tauschii* reaction to *P*. *triticina* is variable with place of host origin (Assefa and Fehrmann, 2000). On the other hand, leaf rust symptoms have never been observed in natural stands of *Aegilops* species including *Ae*. *tauschii* (Anikster et al., 2005; Liu et al., 2014; Gultyaeva et al., 2016). However, infections can develop after inoculation with defined isolates of wheat leaf rust (Anikster et al., 2005), what is in accordance with results obtained in this study.

It was assumed that macroscopic observations of infection rates were not sufficient to determine the exact resistance of hybrid plants. However, general observations were consistent with the microscopic analyses, which were more informative and allowed the visualization of the pathogen structures. The histological investigations of host–pathogen interactions revealed that all analyzed genotypes were infected to a higher level at the seedling stage. Furthermore, a race-specific resistance of the obtained hybrids was observed. Isolate 1 (wxr77) was the least aggressive for all analyzed genotypes, especially at the adult plant stage. It was reported that with the exception of *Lr47* this isolate was virulent against *Lr* genes located on the A-genome, i.e., *Lr10, Lr11, Lr20, Lr28, Lr37, Lr49* (Serfling et al., 2016). Isolates 2 and 3 were determined as virulent for *Lr22a* and *Lr39*, respectively, which was confirmed in present study. According to macroscopic and microscopic results we observed that hybrid plants inoculated at the seedling stage with isolate 3 were less infected in comparison to inoculation with isolates 1 and 2 (**Figures 3, 5**). Similarly, hybrid plants inoculated at the adult plant stage with isolate 2 were characterized by less disease severity in comparison to results obtained with isolate 3. These findings indicate appropriate selection of the *P*. *triticina* isolates and that observed resistance is conferred by both genes introgressed with chromosome 2D. Variations in the resistance were observed also in both Thatcher NILs inoculated with the same isolates. The effectiveness of leaf rust resistance varies, depending on the combination of a particular race-specific *Lr* gene and a corresponding *P*. *triticina* avirulence gene (Bolton et al., 2008). According to Kalia et al. (2017) accessions of *Ae*. *tauschii* exhibiting high APR were found in Azerbaijan, followed by Iran and Afghanistan. The origin of *Ae*. *tauschii* accession D51 used in this study to obtain amphiploids and hybrid plants with triticale is unknown (Majka et al., 2017). Nevertheless, accessions with high APR are likely to carry either race-specific APR gene like *Lr22a* or a combination of two or more minor genes (Hiebert et al., 2007). It is also known that *Lr22a* is expressed only at the adult plant stage, however, the degree of resistance is comparable to *Lr* genes determining high resistance at the seedling stage in contrast to genes like *Lr34*, which confer the slow-rusting type APR (Hiebert et al., 2007). Our results revealed the decreased reaction of hybrid plants at the seedling stage, followed by the increase of resistance in further stages of development giving hint that obtained hybrid plants may especially exhibit APR resistance conferred by *Lr22a* introgressed from *Ae*. *tauschii*. On the basis of the macroscopic and microscopic analyses, this resistance can be determined as additive and race-specific to four selected isolates what is in accordance with previous assumptions (Hiebert et al., 2007; Kalia et al., 2017). So far, the resistance conferred by *Lr22a* was found in only three Canadian cultivars of spring wheat: AC Minto, 5500 HR and 5600 HR or breeding lines with AC Minto in their pedigree. *Lr22a* was introgressed from the AC Minto into susceptible Swiss cultivars CH Campala and CH Rubli with no associated negative effects (Fossati, personal communication). Because of the low exposure to *P*. *triticina*, this gene can be used for pyramiding of leaf rust resistance (Hiebert et al., 2007). The resistance conferred by this gene applied to triticale is reported for the first time in this study.

Seedling resistance gene *Lr39* is linked to the marker *Xgdm35* and the 180 bp fragment obtained for *Ae*. *tauschii* is reported to be indicative for resistance (Singh et al., 2012), whereas *Xgwm296* is localized 2.9 cM from *Lr22a* (Hiebert et al., 2007). In this study, we confirmed the presence of both markers in all fourteen analyzed hybrid genotypes demonstrating their effectiveness in marker-assisted selection. Hence the simultaneous transfer of APR and seedling resistances genes, i.e., *Lr22a* and *Lr39* into breeding lines reduces the vulnerability to a breakdown by virulent races within the leaf rust population which was reported for *Lr39* by Kolmer and Anderson (2011) and other seedling resistances already.

According to macroscopic and microscopic results, we observed no dependence between generation of hybrid plants and resistance. Among four of the most resistant genotypes, two of them were from the earliest generation BC_2_F_4_. It is worth to mention, that DH lines obtained from genotypes 1 and 2 were characterized by the presence of two chromosomes of 2D, whereas genotype 11 is not. The effectiveness of *in vitro* cultures was determined as the number of plants compared to the number of anthers (**Table 2**). The number of green haploid plants ranged from eight for M2DA_1 (0.5 per 100 anthers) to 36 for M2DA_3 (1.6 per 100 anthers), and was low in comparison to previous reports regarding DHs production of triticale, which resulted from 0.4 to 7.9 green plants per 100 anthers (Ślusarkiewicz-Jarzina and Ponitka, 1997, 2003; Ponitka et al., 1999).

## Conclusion

In conclusion, we selected four monosomic 2D addition triticale genotypes highly resistant to *P*. *triticina* infection at all stages of development. The level of resistance was determined in inoculation tests combined with macroscopic and microscopic observations of leaf rust infection. From the selected genotypes, we obtained 26 DH lines among which two lines with doubled additional chromosomes 2D of *Ae*. *tauschii* can be used for further breeding work to increase the agronomic value and the resistance against leaf rust at the seedling and adult plant stages of cultivated triticale.

## Author Contributions

MM initiated project, designed the study, performed the FISH/GISH and molecular marker analysis, and wrote the manuscript. MM, MK, and HW obtained the hybrid plants. AŚ-J produced the DH plants. MM, AS, PC, and FO performed inoculation tests and analyzed the data. All authors agreed to be accountable for all aspects of the work in ensuring that questions related to the accuracy or integrity of any part of the work are appropriately investigated and resolved. All authors contributed and approved the final manuscript.

## Conflict of Interest Statement

The authors declare that the research was conducted in the absence of any commercial or financial relationships that could be construed as a potential conflict of interest.
