# Supplementary material for: Resistance of (Aegilops tauschii × Secale cereale) × Triticosecale Hybrids to Leaf Rust (Puccinia triticina) Determined on the Macroscopic and Microscopic Level
Source: Front Plant Sci. 2018 Sep 26;9:1418. doi: 10.3389/fpls.2018.01418 (PMC6168713; doi:10.3389/fpls.2018.01418)
Supplement: Supplementary file 2 [file Table_2.doc]

**Table S2.** Macroscopic reaction and microscopically visible generation of uredospore pustules of hybrids carrying leaf rust resistance genes and control plants inoculated with single spore isolates at 168 hai - adult plant stage.

| adult plant stage | | | | | | | | |
| --- | --- | --- | --- | --- | --- | --- | --- | --- |
| Genotype | isolate 1 | | isolate 2 | | isolate 3 | | isolate 4 | |
|  | Rating | Colonies >100 spores | Rating | Colonies >100 spores | Rating | Colonies >100 spores | Rating | Colonies >100 spores |
| Hybrid genotype 1 (BC_2_F_4_) | 0.0±0.0 | 0.0±0.0 | 0.0±0.0 | 0.0±0.0 | 1.3±0.3 | 3.3±1.3 | 3.0±0.0 | N/A |
| Hybrid genotype 2 (BC_2_F_4_) | 0.0±0.0 | 0.0±0.0 | 0.0±0.0 | 0.0±0.0 | 0.5±0.3 | 1.0±1.0 | 3.0±0.0 | N/A |
| Hybrid genotype 3 (BC_2_F_5_) | 0.5±0.3 | 0.0±0.0 | 0.0±0.0 | 0.0±0.0 | 2.7±0.3 | 5.3±1.8 | 4.0±0.0 | N/A |
| Hybrid genotype 4 (BC_2_F_5_) | 1.0±0.0 | 0.0±0.0 | 0.7±0.3 | 1.0±0.6 | 1.7±0.9 | 2.7±2.2 | 4.0±0.0 | N/A |
| Hybrid genotype 5 (BC_2_F_5_) | 0.7±0.3 | 0.0±0.0 | 0.7±0.3 | 0.0±0.0 | 3.0±0.0 | 5.3±0.3 | 4.0±0.0 | N/A |
| Hybrid genotype 6 (BC_2_F_5_) | 0.7±0.2 | 0.0±0.0 | 0.5±0.0 | 0.0±0.0 | 2.3±0.3 | 4.7±0.3 | 4.0±0.0 | N/A |
| Hybrid genotype 7 (BC_2_F_5_) | 0.0±0.0 | 0.0±0.0 | 1.7±0.3 | 6.3±1.2 | 1.3±0.3 | 3.0±2.0 | 4.0±0.0 | N/A |
| Hybrid genotype 8 (BC_2_F_5_) | 0.7±0.2 | 0.0±0.0 | 1.3±0.3 | 3.7±0.3 | 3.0±0.0 | 12.3±3.5 | 4.0±0.0 | N/A |
| Hybrid genotype 9 (BC_2_F_5_) | 0.7±0.3 | 0.0±0.0 | 1.7±0.3 | 6.0±1.0 | 2.7±0.3 | 9.0±2.1 | 4.0±0.0 | N/A |
| Hybrid genotype 10 (BC_2_F_6_) | 0.5±0.0 | 0.0±0.0 | 0.7±0.3 | 0.0±0.0 | 0.5±0.0 | 0.0±0.0 | 4.0±0.0 | N/A |
| Hybrid genotype 11 (BC_2_F_6_) | 0.0±0.0 | 0.0±0.0 | 0.3±0.3 | 3.3±0.9 | 1.0±0.0 | 0.0±0.0 | 3.3±0.3 | N/A |
| Hybrid genotype 12 (BC_2_F_6_) | 1.3±0.3 | 0.0±0.0 | 1.3±0.3 | 1.0±0.6 | 2.7±0.3 | 9.0±1.5 | 4.0±0.0 | N/A |
| Hybrid genotype 13 (BC_2_F_6_) | 0.0±0.0 | 0.0±0.0 | 1.3±0.3 | 0.7±0.3 | 2.3±0.3 | 8.7±0.7 | 4.0±0.0 | N/A |
| Hybrid genotype 14 (BC_2_F_6_) | 0.0±0.0 | 0.0±0.0 | 0.5±0.3 | 2.0±0.6 | 0.0±0.0 | 0.0±0.0 | 3.3±0.3 | N/A |
| Hybrid genotype 15 (BC_2_F_7_) | 0.5±0.3 | 0.0±0.0 | 1.7±0.3 | 5.3±1.2 | 1.7±0.3 | 4.0±2.1 | 4.0±0.0 | N/A |
| Hybrid genotype 16 (BC_2_F_7_) | 1.0±0.0 | 0.0±0.0 | 1.7±0.3 | 5.7±0.9 | 2.7±0.3 | 14.3±1.3 | 4.0±0.0 | N/A |
| *Ae. tauschii* | 3.0±0.0 | 9.0±0.6 | 3.0±0.0 | 11.0±1.2 | 3.0±0.0 | 12.3±1.5 | 3.3±0.3 | N/A |
| *AtSc* amphiploid | 0.0±0.0 | 0.0±0.0 | 1.0±0.0 | 4.3±0.3 | 1.7±0.3 | 2.3±1.5 | 2.7±0.3 | N/A |
| Triticale cv. Bogo | 1.0±0.0 | 0.0±0.0 | 1.0±0.0 | 2.0±0.6 | 1.7±0.3 | 0.7±0.7 | 4.0±0.0 | N/A |
| Wheat cv. Thatcher | 3.0±0.0 | 12.3±0.7 | 3.0±0.0 | 10.7±0.7 | 3.0±0.0 | 12.3±0.9 | 4.0±0.0 | N/A |
| Tc+*Lr22a* | 2.3±0.3 | 9.7±1.2 | 3.0±0.0 | 16.0±2.6 | 2.7±0.3 | 8.7±0.9 | 3.0±0.0 | N/A |
| Tc+*Lr39* | 0.0±0.0 | 0.0±0.0 | 1.0±0.0 | 4.0±0.6 | 2.0±0.0 | 7.0±0.6 | 1.3±0.3 | N/A |
| Wheat cv. Borenos | 3.0±0.0 | 15.0±1.7 | 3.0±0.0 | 8.7±0.7 | 3.0±0.0 | 22.3±1.5 | N/A | N/A |
